# The United States Pharmacopea

**Published:** 1877-03-20

**Authors:** 


THE UNITED STATES PHARMACOPEA.—We are in receipt of a pamphlet entitled the *United States Pharmacopea and the American Medical Association*, in which are important and forcible reasons against the proposition of Dr. Squibb for taking the work from the National Convention and placing it in the hands of the American Medical Association.

The Pamphlet will be sent to any Physician who will send his address and a stamp to Dr. H. C. Wood, 1631 Arch street, Philadelphia.

